# Epigenetic clocks and programmatic aging

**DOI:** 10.1016/j.arr.2024.102546

**Published:** 2024-10-15

**Authors:** David Gems, Roop Singh Virk, João Pedro de Magalhaes

**Affiliations:** 1 Institute of Healthy Ageing, and Research Department of Genetics, Evolution and Environment, University College London, London, WC1E 6BT, United Kingdom; 2 Genomics of Ageing and Rejuvenation Lab, Institute of Inflammation and Ageing, University of Birmingham, B15 2WB, United Kingdom

**Keywords:** aging, development, epigenetics, hyperfunction, methylation clocks, programmatic theory

## Abstract

The last decade has seen remarkable progress in the characterization of methylation clocks that can serve as indicators of biological age in humans and many other mammalian species. While the biological processes of aging that underlie these clocks have remained unclear, several clues have pointed to a link to developmental mechanisms. These include the presence in the vicinity of clock CpG sites of genes that specify development, including those of the Hox (homeobox) and polycomb classes. Here we discuss how recent advances in programmatic theories of aging provide a framework within which methylation clocks can be understood as part of a developmental process of aging. This includes how such clocks evolve, how developmental mechanisms cause aging, and how they give rise to late-life disease. The combination of ideas from evolutionary biology, biogerontology and developmental biology open a path to a new discipline, that of *developmental gerontology* (*devo-gero*). Drawing on the properties of methylation clocks, we offer several new hypotheses that exemplify devo-gero thinking. We suggest that polycomb controls a trade-off between earlier developmental fidelity and later developmental plasticity. We also propose the existence of an evolutionarily-conserved *developmental sequence* spanning ontogenesis, adult development and aging, that both constrains and determines the evolution of aging.

## 1 Introduction

“*an understanding of maximal life span and age at maturity in mammals will require a merging of physiological, ecological, and evolutionary understandings*” (Prothero, 1993)

Epigenetic alterations can be defined as chemical modifications to DNA or to chromatin structure that lead to changes in gene expression and phenotype, that persist across time or generations, without involving alteration of the DNA sequence. Many types of epigenetic alteration have been described, including DNA methylation and histone modifications as well as gene regulation by non-coding RNAs. The idea that epigenetic changes, particularly DNA methylation, contribute to aging has long been debated (Johnson et al., 2012; Sedivy et al., 2008). Indeed, a wide array of epigenetic changes with age have been described, and life-extending interventions like dietary restriction modulate, and might act partly via, epigenetic processes (Pal and Tyler, 2016).

In recent years, breakthroughs in developing epigenetic clocks that track biological age, late-life health and mortality have not only yielded biomarkers of aging, but have also demonstrated a close correlation between certain methylation changes and aging (Bocklandt et al., 2011; Hannum et al., 2013; Horvath, 2013; Raj and Horvath, 2020). One detail that has been underemphasized is the fact that epigenetic clocks predict age across the entire life course, from early embryogenesis onwards (Horvath, 2013; Kerepesi et al., 2021). The strong correlations between epigenetic changes during development and aging suggest that developmental mechanisms somehow play a role in driving the aging process.

This progress in work on epigenetic clocks coincides with the emergence of a broader picture of programmatic aging, including an ultimate proximate account that integrates evolutionary and mechanistic theories. Programmatic theories of aging have a long history dating back to the 19^th^ century (de Magalhaes and Church, 2005), but have seen major advances in recent years (de Magalhaes, 2023; Gems, 2022).

According to evolutionary theory, genes exhibiting antagonistic pleiotropy, that are beneficial early in life yet detrimental later on, may be favored by natural selection, causing aging (Williams, 1957). In the context of programmatic theories, the basic hypothesis is that developmental gene action and processes that are beneficial earlier in life continue later in life, in a futile fashion, becoming detrimental and pathogenic (de Magalhaes, 2012).

The programmatic theory of aging (sometimes also referred to as the developmental theory of aging, or the hyperfunction theory) is distinct from the disposable soma theory (Kirkwood, 1977), an earlier ultimate proximate theory. However, the former is, arguably, better supported empirically (Blagosklonny, 2007; Gems and Kern, 2024; Maklakov and Chapman, 2019). Here we explore how recent discoveries about methylation clocks cohere with the developmental theory of aging. We present several new perspectives, questions, and speculations arising from the cross-referencing of these two subjects, and argue for the timeliness of interdisciplinary integration of biogerontology with developmental biology. Central to these ideas is the hypothesis that epigenetic changes occurring throughout life history (including ontogenesis and senescence) are part of wider developmental changes.

## 2 Methylation clocks

Although subtle deteriorative changes can be detected in early life (including during fetal development, as in the fatty streak precursors of vascular atheroma formation) (Napoli et al., 1999), most senescent phenotypes are observed in adulthood (Niccoli and Partridge, 2012). Comparing different mammalian species, such phenotypes, including the diverse diseases of aging, are strikingly similar yet develop at very different rates and appear over different age ranges. For example, mice and rats age about 20 to 30 times faster than humans, and experience senescence (including greying of hair, cataracts and cancer) at ages at which humans are still in infancy.

Given the gradual and, to some degree, predictable patterns observed in aging phenotypes in humans and many animal models, whatever mechanisms drive aging must involve gradual changes across tissues with age, as well as distinct rates of changes in species with different lifespans. In other words, there must be gradual, progressive biological changes whose rate determines the timing of onset of the manifold functional and degenerative changes that occur during aging, including the development of multiple age-related diseases. This suggests the presence of timer mechanisms of some sort that specify the rate of senescence - mechanisms that could serve as biomarkers of aging.

### 2.1 Horvath's discovery of a multi-tissue methylation clock

The development by Steve Horvath of a human multi-tissue predictor of age using methylation signatures, initially based on 353 CpG sites (Horvath, 2013), was a major breakthrough in biogerontology. DNA methylation (DNAm) age has since then been widely used as a biomarker of aging, and a variety of other epigenetic clocks have also been devised, including GrimAge, GrimAge2, and DNAm PhenoAge (Field et al., 2018; Galkin et al., 2020; Horvath and Raj, 2018; Noroozi et al., 2021; Simpson and Chandra, 2021).

A notable feature of the Horvath clock is that it ticks from shortly after conception all the way until old age. This clock is also remarkable because it can be applied to a wide range of different tissues; exceptions include the female breast which appears older than other tissues, and the cerebellum which appears younger (Horvath and Raj, 2018). The synchrony of DNAm age across tissues is surprising, particularly given the very different proliferation histories of tissues such as the brain, gut, and blood cells.

Several lines of evidence support the view that epigenetic clocks can serve as bona fide biomarkers of aging. For example, advancing estimated epigenetic age has been shown to be decelerated by treatments that slow aging in mice, such as dietary restriction, inhibition of growth hormone and mTOR signaling, and heterochronic heterobiosis (Horvath and Raj, 2018; Zhang et al., 2023). Moreover, various conditions associated with faster human aging can increase estimated epigenetic age, including obesity, frailty, Down syndrome and Werner syndrome (Horvath and Raj, 2018).

The mechanisms underpinning the various epigenetic clocks remain a subject of debate. They are not, seemingly, a measure of cell proliferation, cellular senescence, telomere shortening or genomic instability. One major open question is which specific cell types contribute to epigenetic clocks. Stem cells have been proposed as major contributors to the epigenetic clock, suggesting the occurrence of slow changes with age in the stem cell activity that maintains tissue homeostasis (Raj and Horvath, 2020). This hypothesis is supported by results of in vitro studies showing that stem cell composition influences epigenetic clocks (Kabacik et al., 2022). Raj and Horvath have also suggested that clock age reflects changes in epigenetic heterogeneity, possibly affecting only a small percentage of cells (Raj and Horvath, 2020).

As mentioned, the epigenetic clock appears to track developmental processes (de Magalhaes, 2023; Horvath and Raj, 2018; Raj and Horvath, 2020). Yet though the clock starts ticking shortly after conception, and at a rate that correlates with growth profiles, the similarity of DNAm age estimates in proliferative and non-proliferative tissue suggests that it is not a measure of cell proliferation or, seemingly, differentiation (although correlations between cell proliferation and epigenetic clocks have been observed in some cell types in culture) (Horvath and Raj, 2018).

A notable early observation by Horvath was that induced pluripotent cells and embryonic cells have an epigenetic age of zero (Horvath, 2013). Epigenetic age increases as differentiated cells (fibroblasts) proliferate in vitro, but not in embryonic cells until they differentiate (Kabacik et al., 2022). More recently, it has been shown that partial and transient reprogramming of cells can lower their estimated epigenetic age, consistent with cellular rejuvenation (Sarkar et al., 2020; Simpson et al., 2021). Thus, there is evidence that cellular reprogramming (putative rejuvenation) can reverse age-related changes in methylation, at least to some degree.

### 2.2 A universal mammalian clock

Perhaps even more remarkable than the development of the Horvath multi-tissue clock is the recent discovery that methylation clocks are conserved across mammalian species with major differences in lifespan. Using a large dataset from 185 mammalian species, a universal clock was developed that provides an accurate estimate of age in both eutherian and marsupial mammals (Lu et al., 2023).

Another, recent groundbreaking study led by Horvath developed methylation-based models that accurately predict the maximum lifespan (*DNAm maximum lifespan*) (*R* = 0.89), gestational time (*R* = 0.96), and age at sexual maturity (*R* = 0.85) of mammalian species (Li et al., 2024a). These methylation signatures do not necessarily change with age; rather they are set early in life and, remarkably, predict lifespan across different species; a second recent study has also linked methylation rate to maximum lifespan across species (Crofts et al., 2024). Given the strong correlation between the relative timing of life history traits in mammals (de Magalhaes et al., 2007), these methylation signatures predict the overall pace of life history events, from birth to death. Notably, the lifespan of vertebrate species is also a function of CpG density in gene promoters (Budd et al., 2024; Mayne et al., 2019; McLain and Faulk, 2018), hinting at a possible genetic basis for DNAm maximum lifespan, and also for life history rate evolution.

### 2.3 What is the biology underlying the clock?

Although epigenetic clocks have generated great interest in the field of biogerontology, and beyond, their underlying biology remains poorly understood. Theories about the relationship between methylation clocks and the underlying aging process fall broadly into two categories. First, earlier theories positing that methylation clocks and epigenetic age-related changes in some way reflect accumulation of molecular damage or dysfunction. Second, more recent theories that view epigenetic changes as part of developmental changes involved in both ontogenesis and aging. These interpretations, which are not mutually exclusive, are discussed next.

#### 2.3.1 Damage accumulation clocks

When Horvath published his original clock a decade ago, a widely held assumption was that aging is predominantly the result of accumulation of stochastic molecular damage. In this vein, Horvath suggested a possible mechanistic basis for the DNAm clock: that methylation changes reflect the activity of a maintenance system that protects the epigenome from damage (Horvath, 2013). The simpler theory that DNAm Age is a function of accumulated damage to chromatin (including DNA damage) is still widely entertained (Benayoun et al., 2015; Moqri et al., 2023; Soto-Palma et al., 2022; Yang et al., 2023).

DNAm Age may well be secondary to (i.e. causally downstream of) other changes (Field et al., 2018). Given that somatic DNA damage accumulates with advancing age, it is plausible that this contributes to some degree to age-related epigenetic change, but its relative importance remains unclear. For example, impaired DNA repair leads to epigenome instability in human progeroid syndromes, and epigenetic modifications also seem to be important for DNA damage repair (Soto-Palma et al., 2022). Of note, experiments in mice have shown that DNA damage-induced disruption of the epigenome can accelerate DNAm Age and aging phenotypes (Yang et al., 2023), although whether the epigenetic changes cause the premature aging phenotypes is unclear. Another noteworthy recent finding is an association between mutations at CpG sites and methylation patterns that are predictive of age (Koch et al., 2023). In conclusion, the hypothesis that DNA damage is a determinant of DNAm Age currently remains viable but awaits robust proof.

#### 2.3.2 A developmental clock

Another possibility is that epigenetic clocks track changes during adulthood that are developmental in nature. But what does it mean to speak of developmental processes occurring during adulthood? An obstacle to answering this precisely is the lack of clear terminology relating to the term “developmental”. In very broad terms, two sorts of developmental process can be distinguished, each of which involves growth, differentiation, and morphogenesis.

The first is ontogenesis, the process by which a zygote develops into a sexually mature adult. The second includes developmental processes of maturity, that occur after ontogenesis, including global developmental changes (e.g. during post-maturational growth) and also more localized changes. Among these are developmental changes linked to reproduction, particularly in females (e.g. changes to mammary and uterine tissue during the oestrus cycle, and diverse changes during pregnancy and lactation), growth and atrophy of muscle and adipose tissue in response to changes in levels of food consumption and exercise, and diverse processes of tissue repair and immunity, from wound healing and bone fracture repair to clearance of infection by the acquired and innate immune systems.

**Fig. 1:**
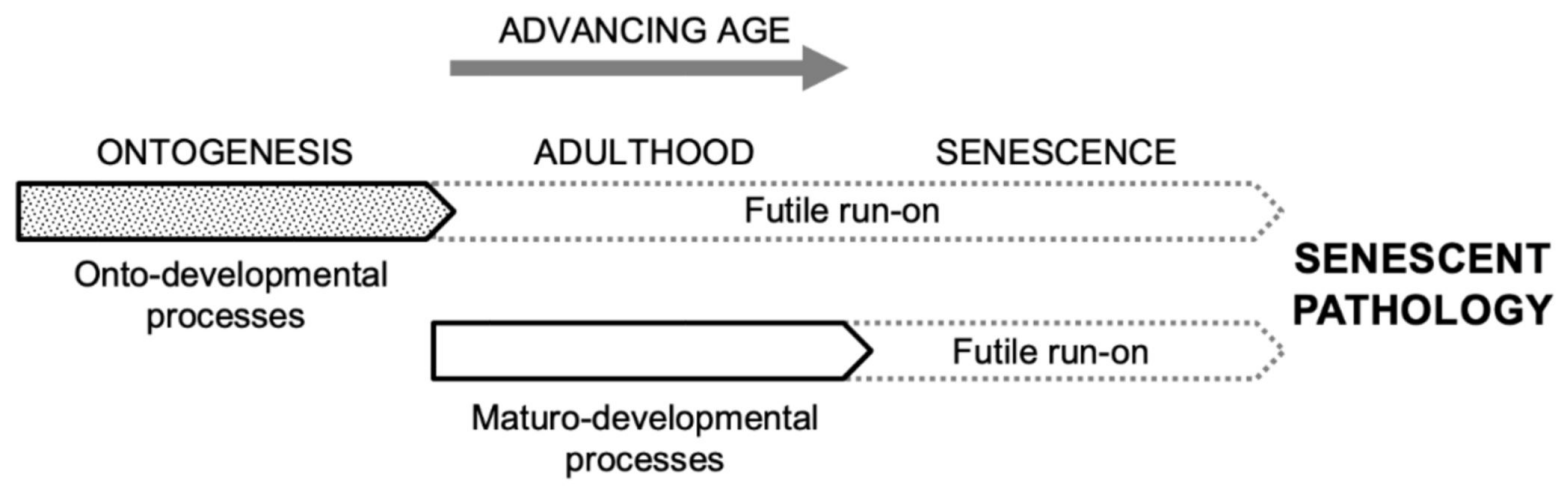
Onto-developmental and maturo-developmental process and their run-on in development, maturity and aging. Simplified hypothetical scheme, omitting a number of details, as follows. Some adaptive ontogenetic processes may continue on during early adulthood. Some maturo-developmental processes (wound healing, immunity) will also be operative prior to adulthood, and also in later life in parallel to their futile, quasi-programmed derivatives.

To clearly distinguish these two classes of developmental process, we suggest the respective terms *onto-developmental* and *maturo-developmental*. In principle, there are several types of developmental process active during adulthood that methylation clocks could track. One is adaptive maturo-developmental changes (as described in the previous paragraph). Another, as predicted by the programmatic theory, is non-adaptive, futile run-on of ontogenetic processes in adulthood, as in the continuation of lens growth that causes presbyopia (inability of the eye to focus on nearby objects) (Strenk et al., 2005). However, a second prediction of the programmatic theory is that non-adaptive, futile activation also occurs with maturo-developmental processes, leading e.g. from useful acute inflammation to the chronic sterile inflammation that contributes to many late-life diseases (e.g. via fibrogenesis) (Figure 1).

The fact that the Horvath clock ticks throughout ontogenesis, and at a much faster rate than after puberty (Raj and Horvath, 2020), is consistent with the idea that it tracks developmental processes. Notably, comparison in mice of methylation changes to CpG sites during ontogenesis and adulthood support the view that the latter are, to some extent, a continuation of the former, rather than a product of a deteriorative process that starts in adulthood (Takasugi, 2011).

Further evidence of a link between epigenetic clocks and development has emerged from examination of genes in the region of clock methylation sites. In the universal mammalian clock, CpG sites that change with age were enriched for polycomb repressive complex 2 (PRC2)-binding locations (Lu et al., 2023). Similarly, lifespan-related CpGs are often located in regions that are transcriptionally repressed or at least bound by PRC2 proteins (Moqri et al., 2024), and hypomethylated in long-lived species (Li et al., 2024a).

Polycomb group proteins are best known for their function in silencing Hox genes during ontogenesis through alteration of chromatin structure, and as such are fundamental regulators of pattern formation during development (Li et al., 2024b). Considering the link between methylation clocks and PRC2 function, that is conserved across mammals, Horvath and his collaborators deduced that methylation of genes involved in development may be a key mechanism linking growth, development and aging, concluding that “aging is evolutionarily conserved and intertwined with developmental processes across all mammals” (Lu et al., 2023).

It is important to emphasize here that epigenetic clocks reflect only a very small subset of methylation changes that occur with aging. Nonetheless, the fact that such accurate clocks can be built across tissues and species is remarkable, and underscores the deterministic nature of aging changes, even across species with vastly different lifespans. Development rate and lifespan are strongly correlated across mammalian species (de Magalhaes et al., 2007). This being so, if the epigenetic clocks are part of developmental processes that run faster in shorter-lived species, this could explain the accuracy of the clocks across species (de Magalhaes, 2023).

## 3 Understanding epigenetic aging within the bigger picture

Understanding how methylation clocks fit into the wider aging process would require an explanatory framework for the latter. Unfortunately, there is currently little consensus about the causes of aging. The much-cited hallmarks of aging (López-Otín et al., 2013; López-Otín et al., 2023), while a handy point of reference for the field, is not an explanatory framework in any real sense (Gems and de Magalhaes, 2021). Since 2005, we and others have been attempting to develop such a framework, drawing on accumulated findings in the field, and integrating evolutionary and mechanistic theories of aging - an evolutionary physiology approach (Arnold and Rose, 2023; Blagosklonny, 2006; de Magalhaes and Church, 2005; de Magalhaes, 2023; Gems, 2022; Gems and de la Guardia, 2013; Lemaître et al., 2024; Maklakov and Chapman, 2019; Slade et al., 2024).

This emerging framework provides potential explanations for the evolution of methylation clocks, the developmental mechanisms within which they act, and how such mechanisms give rise to organismal senescence (including late-life disease). In addition, the emerging link between methylation clocks and developmental mechanisms, and particularly the recently discovered involvement of polycomb proteins, draw attention to the potential value of integrating knowledge from biogerontology and developmental biology to understand mechanisms of aging.

In the remainder of this article, we briefly review how combining evolutionary theory and mechanistic biogerontology (evolutionary physiology) can provide an explanation of epigenetic aging. We then explore links between theories of aging and ideas from developmental biology, in a *developmental gerontology (devo-gero)* approach (or *devo-aging*, to use another recently coined term) (Silva-García, 2023).

### 3.1 How aging evolves

With respect to understanding aging and late-life disease, one certainty is that their main, ultimate cause is the process of evolution (Arnold and Rose, 2023). The aging rate and maximum lifespan of any given animal species is largely set by its genome, which is the product of evolution. However, aging and limited lifespan is not an adaptation; rather, it is a non-adaptive by-product of the evolutionary process.

Because more young adult animals contribute to the next generation than older adults, the force of natural selection declines with age. Consequently, genetic variants that are detrimental early in life, in particular those prior to reproduction, will experience stronger selection against them than those that are detrimental later in life. This leads to the accumulation of genetic variants with late-acting detrimental effects, which cause aging (Medawar, 1952).

As described above, some genes that promote fitness earlier in life are detrimental later in life, exhibiting the property of antagonistic pleiotropy (AP) (Williams, 1957). Many genes have now been identified with this property, enhancing early life fitness and causing late-life disease, in animal species and humans (Austad and Hoffman, 2018; Gems and Kern, 2024). The ubiquity of genes exhibiting AP reflects the major role of “buy now, pay later”-type evolutionary trade-offs as a major underlying cause of aging.

### 3.2 Integrating evolutionary and mechanistic theories of aging

The antagonistic pleiotropy theory explains how genes evolve that cause aging. However, it says little about the biological mechanisms of aging that such genes specify, i.e. the nature of the aging process itself. Critical to understanding the biology of aging is development of accounts that integrate evolutionary (ultimate) and mechanistic (proximate) explanations of aging. Two such ultimate-proximate (or evolutionary physiology) theories are the disposable soma theory and the programmatic theory. That the latter currently appears more plausible (or, at least, more widely applicable) has been argued elsewhere (Blagosklonny, 2007; Gems and Kern, 2024).

The programmatic theory incorporates several new concepts and definitions. In the broadest sense, the theory argues that aging can be understood as the product of the genome, as a phenotype corresponding to a genotype. This contrasts with the idea that aging is largely a product of molecular damage or other processes of passive deterioration. The theory views aging as *programmatic* but not *programmed*. Here the word programmed can be taken to mean that aging is genetically determined, which is true; or that aging is an adaptation, which is false. To disambiguate this, the adjective *quasi-programmed* was introduced (Blagosklonny, 2006), whose meaning is similar to that of the word programmatic. However, the related noun *quasi-program* has no equivalent derivation from programmatic, and is a useful term to describe entities that cause late-life disease.

The programmatic theory includes the idea that developmental mechanisms, resulting from normal gene action, play a role on aging. It also incorporates an interpretation of how AP works in terms of biological mechanisms. This is based on the understanding that biological systems are subject to a high degree of biological constraint (Gems and Kern, 2024). The type of constrained system involved is one where the fitness benefits from several traits cannot all be maximized due to functional interconnections between them. For example, the product of the AP gene *ORL1* (lectin-like low-density lipoprotein receptor 1) appears to promote immune defense by binding bacterial cell wall proteins, but also promote atherosclerosis by binding oxidized low density lipoprotein (LDL) in endothelial cells (Predazzi et al., 2013). Here binding properties to one target (bacterial cell wall proteins) are inseparable from those to another (oxidized LDL).

In such cases evolutionary optimization of cost and benefit can lead in later life to levels of function that are either excessive (*hyperfunction*) or insufficient (*hypofunction*) with respect to one of the coupled traits, thereby contributing to the development of senescent pathology (Gems and Kern, 2024). It has been argued that evolved, wild-type hyperfunction in mTOR signaling in particular is more important as a primary cause of aging than molecular damage accumulation (Blagosklonny, 2008).

Regarding epigenetic aging, one hypothesis is that the Horvath clock reflects the progression of developmental processes in adulthood (de Magalhaes, 2023). As cells divide and differentiate during development, which is coordinated by the genetic program encoded in the DNA, epigenetic changes occur in cells. In adulthood, a subset of onto- or maturo-developmental processes continue on, reflecting AP in developmental genes. As part of these developmental changes, cells continue to change epigenetically, contributing to aging phenotypes, and reflected in the continued ticking of the Horvath clock.

### 3.3 Towards a new field of developmental gerontology (devo-gero)

An exciting prospect presented by the convergence of advances relating to methylation clocks and in theories of aging is that of further convergence with the field of development biology. This includes both integration of mechanisms of development and senescence, and ultimate proximate accounts that encompass both development and senescence. With the hope of provoking thought and discussion in a devo-gero vein, in the remainder of this review we present several new and supported hypotheses relating to this nascent subject area.

Let us first consider the integration of mechanisms of development and senescence. If developmental mechanisms are determinative of aging, then the highly advanced science of developmental biology (including developmental genetics) should represent a treasure trove of information that can be repurposed to understand the biology of senescence. A case in point here is polycomb proteins, whose role in the biology of development has been studied for many decades (Li et al., 2024b). The extensive existing knowledge should provide clues as to the significance of the link between methylation clocks, polycomb proteins and aging.

#### 3.3.1 Onto-developmental fidelity vs maturo-developmental plasticity?

During the course of adulthood (including aging) in animals ranging from mammals to amphibians, chromatin accessibility is progressively increased (Bozukova et al., 2022; Morandini et al., 2024). This particularly affects regulatory regions associated with polycomb-group (PcG) genes and PcG protein target genes in most if not all cell types. Based on several considerations we suggest the hypothesis that this reflects the shift from onto-developmental to maturo-developmental processes.

Arguably, a difference between these two types of process is that in ontogenesis, fidelity in developmental processes is at a premium to ensure formation of an optimally functional body. By contrast, maturo-developmental processes confer adaptability (phenotypic plasticity) during adulthood. The latter is important to enable the organism, once generated by its ontogenetic program, to be able to adapt and survive in the face of a changeable environment (including variation in reproductive opportunity and food availability, and incidence of injury and infection). To put it simply, we suggest that ontogenesis aims to produce an organism more to specification, while maturity chops and changes the organism to optimize performance, in a developmental equivalent of behavior (though of course ontogenesis can also exhibit some degree of plasticity) (developmental plasticity).

While both fidelity and plasticity in developmental programs are advantageous, these are, to some extent, mutually exclusive properties. This means that their respective benefits cannot be simultaneously maximized, i.e. that these two properties are subject to biological constraint. This creates the conditions for trade-offs between developmental fidelity and adaptability, and AP in genes affecting these trade-offs (Gems and Kern, 2024). We suggest that at the end of ontogenesis, when developmental fidelity is no longer at such a premium, selection for this characteristic weakens, and that for adaptability strengthens.

**Fig. 2:**
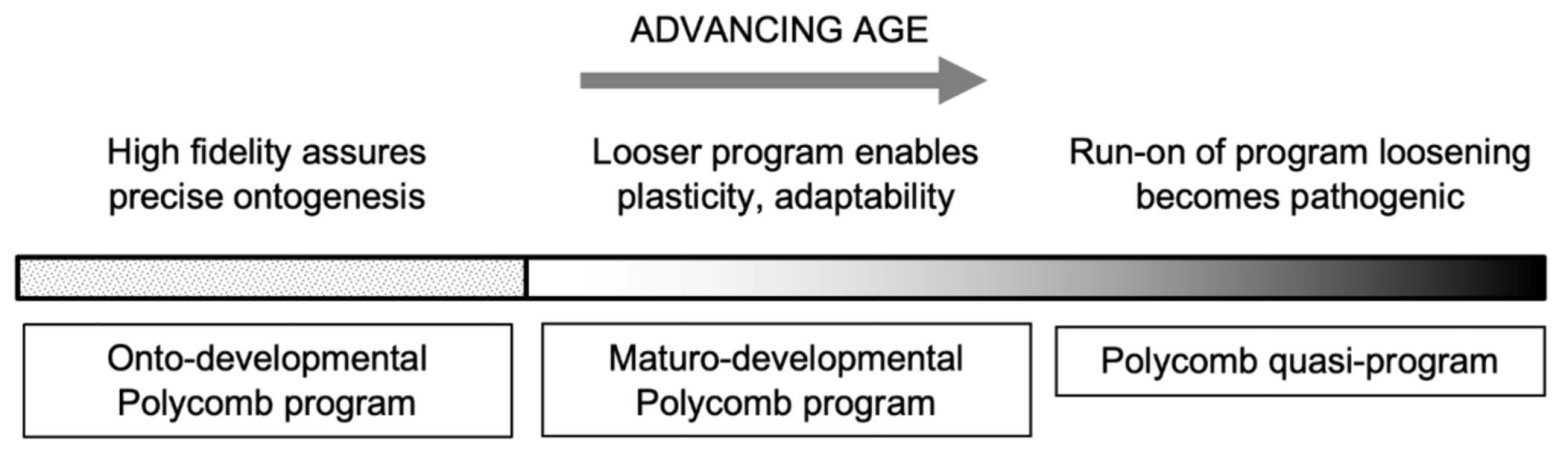
Onto-developmental fidelity vs maturo-developmental plasticity hypothesis. Developmental fidelity is critical during ontogenesis, but less so thereafter. Completion reduces selection for fidelity but increases that for plasticity (adaptability). An adult shift towards the latter is facilitated by Polycomb. In later life, this leads to pathogenic quasi-programs that promote late-life diseases. Run-on of epigenetic loosening might contribute to the late-life explosion of retrotransposition, and the consequent increase in systemic inflammation (De Cecco et al., 2019; Gorbunova et al., 2014).

We postulate that the age-associated increases in chromatin accessibility, and gradual change in the epigenomic state of PcG-associated genes, promotes developmental plasticity, which facilitates adaptive maturo-developmental change. During adulthood prior to senescence, such maturational changes are adaptive, supporting reproduction, tissue homeostasis, wound and bone fracture healing, and immunity, among other processes. However, due to the weakening force of natural selection in later life, AP alleles are present that promote run-on of the progressive increase in plasticity, leading to loss of developmental control (Figure 2) (Jung and Pfeifer, 2015). This account to an extent draws on the recently proposed hypothesis that breakdown of epigenetic polycomb mechanisms leads to maladaptive “phenotypic pliancy” (i.e. divergence from the norm) that promotes cancer development (Lambros et al., 2023). But in our account such pliancy is initially adaptive.

A prediction of this theory is that late-life diseases will tend to occur at sites of maturo-developmental plasticity. One possible example relates to breast cancer. Epithelial tissue within the breast shows maturo-developmental plasticity that supports lactational function. It was recently reported that over the course of time during adulthood, the methylome of healthy proliferating breast luminal epithelial tissues increasingly resembles that of breast cancer (Sayaman et al., 2022; Senapati et al., 2023). In particular, hypermethylation of polycomb repressive complex 2 target genes in CpG islands occurs in both contexts. This is a potential example of a maturo-developmental process displaying AP. Here, a pro-plasticity program facilitates developmental changes that take place during lactation. In line with programmatic theory, such beneficial plasticity runs on in later-life into quasi-programmed maturo-developmental changes that promote carcinogenesis. This principle could help to explain why reproductive organs tend to have a higher mass-normalized cancer incidence (Silva et al., 2011). A further possibility is that this accounts for the greater DNAm age of breast tissue (Horvath and Raj, 2018). Notably, age-changes in polycomb contribute to senescent changes in the fruit fly *Drosophila melanogaster*, suggesting a possible early evolutionary appearance of this principle (Tauc et al., 2021).

Whether correct or not, this speculative hypothesis illustrates a style of thinking (devo-gero) that combines developmental, gerontological and also etiological (disease biology) perspectives. The latter is particularly important, since what the study of the biology of aging most urgently needs to provide is explanations for the causes of late-life diseases.

#### 3.3.2 A developmental sequence constraining life history evolution?

Next, we present a new and more general hypothesis about aging of an ultimate proximate nature, drawing on recent findings relating to methylation clocks, and developmental perspectives. We will introduce this by way of a minor elaboration of the concept of aging clocks.

The notion of methylation clocks implicitly involves an analogy with the clocks whose use is part of our daily life. To make this more explicit, the type of clock that is most useful to draw comparisons with is not the sort used to tell the time of day, but rather something akin to instruments that measure a single time sequence. An example here is an egg timer (or kitchen timer) of the old-fashioned type, with a round face bearing graduations (time interval marks) on the circumference, and an adjustable dial bearing a timer hand. In this form of countdown device, two elements determine the duration of the set time: the distance (measured by the number of graduations) that the timer hand has to pass, and the speed with which the hand moves (the tick rate).

Methylation clock analysis has shown that the tick rate varies across the life course, and also that interventions that extend or reduce lifespan can slow down or speed up the tick rate (Raj and Horvath, 2020). With the egg timer analogy in mind: what remains undefined is the biological entity that corresponds to the limited sequence of graduations on the timer face. Here we present a hypothesis about the nature of that entity, as it exists in mammals.

**Fig. 3:**
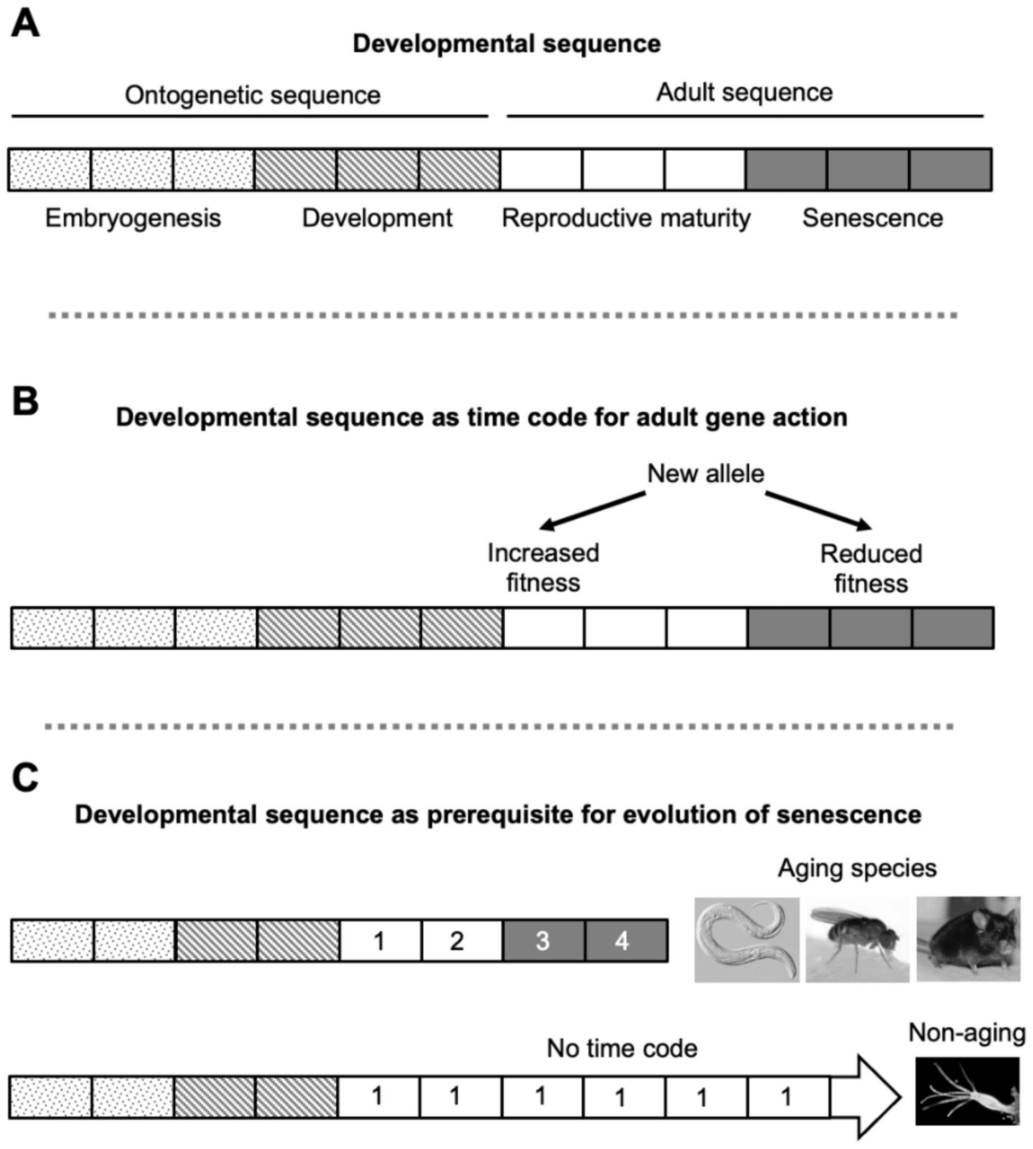
The developmental sequence hypothesis. A, This hypothetical entity originates at conception and ends in at death from old age. The sequence has two successive parts, the ontogenetic developmental sequence (ontogenetic sequence) from conception to sexual maturity and the adult developmental sequence (adult or maturo-developmental sequence) from sexual maturity to death from senescence. B, The developmental sequence provides a time code that allows trade-offs between earlier and later gene effects to evolve. C, In the absence of the developmental sequence, such trade-offs cannot evolve, and organisms remain in a non-aging state, as in *Hydra vulgaris*; notably, a study that attempted to develop a methylation clock for an amphibian (the axolotl, *Ambystoma mexicanum*) observed methylation changes during ontogenesis but not adulthood (Haluza et al., 2024).

We suggest that there exists a series of developmental stages, or *developmental sequence*, that originates at conception and ends at death from old age, and is evolutionarily conserved across mammalian species. This developmental sequence includes earlier ontogenetic stages and later maturo-developmental stages (Figure 3A).

In principle, aging rate could change due to alteration in either the developmental sequence length or the speed at which it runs. The latter will be referred to here as the *play rate*, to distinguish it from the methylation clock tick rate (discussed further below). Given the high degree of conservation of ontogenesis across mammalian species, it is evident that differences in the duration of this process are, in the main, attributable to changes in play rate (development rate) rather than changes in ontogenetic sequence length. This suggests that the same may be true of variation in the length of the adult developmental sequence. If correct, this would imply that differences in lifespans between species is largely a function of different play rates of a relatively invariant developmental sequence. This is potentially of relevance to the evolution of aging, as follows.

A surprising feature of mammalian life history is that across species the ratio of adult lifespan to time from conception to sexual maturity is approximately constant (Charnov, 1993; de Magalhaes et al., 2007) (J.P. de Magalhaes, unpublished). This is an example of isometry, a term usually applied to body part size, where the relative proportions of different structures remain constant. For example, due to bilateral symmetry the ratio of the size of the left ear to the right is 1. In terms of lifespan pre- and post-sexual maturity, mammals show approximate isometry (here temporal rather than spatial).

A standard explanation of the cause of such isometry between developmental span and adult lifespan is that it is due to effects of the late-life selection shadow on both (Charnov, 1993; Harvey and Purvis, 1999; Promislow, 1993). Yet although it is easy to see how an increased selection shadow could lead to shorter development time and lifespan, it is less obvious why selection for increased longevity should cause a proportional lengthening of the developmental period (Dilman, 1994). For example, one of the longest-lived rodents, the naked mole rat (*Heterocephalus glaber*) has a gestation time of ~90 days (Roellig et al., 2011), compared to only ~20 days in the house mouse, a similar-sized rodent, as well as features of extremely protracted postnatal development (Penz et al., 2015). Why would selection for adult longevity in naked mole rats have led to such a slow development rate? In line with this, in classic selection experiments using *D. melanogaster*, selection for longer lifespan led to prolonged development (Chippindale et al., 1994), while that for faster development led to shorter lifespan (Chippindale et al., 1997; Chippindale et al., 2004).

We postulate that an additional determinant may contribute to the isometry between adult span and developmental span: biological constraint imposed by the presence of the invariant developmental sequence. According to this model, critically: the sequence is constrained, but the overall play rate is not. Here, due to the presence of biological constraint, selection for longevity (to support later reproduction) will also lead to a coupled retardation of development rate. The latter may be understood as an evolutionary spandrel, i.e. a non-adaptive by-product of the selection of an adaptive trait (Gems and Kern, 2024; Gould and Lewontin, 1979).

To be precise about a detail of this model: we propose here that the play rate of the developmental sequence is not constrained. This is with reference to the egg-timer model, not the tick rate of the methylation clock as described by Horvath. The rate of the latter varies greatly across the course of the life history, and its pattern of ups and downs is likely to be tightly coupled to the developmental sequence, and therefore constrained. However, the relative speed of the entire developmental program - the play rate - is, we postulate, plastic. Possibly, it is the setting of this play rate that is captured by Horvath’s cytosine methylation signatures that predict differences in gestation time, age at sexual maturity, and maximum lifespan between mammalian species (Li et al., 2024a).

An intriguing possibility is that the adult developmental sequence provides a timeline or time code (as used to synchronize sound and image in celluloid film) that enables the timing of beneficial and harmful gene effects to be reliably specified (Figure 3B). Arguably, the more precisely that the timing of the costs and benefits of “buy-now, pay-later”-type trade-offs can be defined, the greater the possibility to reliably reap their benefits. In other words, the presence of the developmental sequence, and the time code it provides, may be a precondition for aging to evolve. The absence of an adult developmental sequence might explain the failure of senescence to evolve in non-aging species such as *Hydra vulgaris* (Figure 3C). In a similar fashion, uncertainty about the future is anathema to business.

Recent work on the link between age-changes in epigenetic state and effects of genetic polymorphisms on late-life disease have led to the proposition that there exists a “central trajectory for epigenetic state that reflects innate aging processes” (Boyle et al., 2012; Richard and Capellini, 2021). This idea is readily reconciled with the developmental sequence hypothesis.

An intriguing possibility is that of identifying and manipulating the mechanism controlling the developmental sequence play rate, that is subject to evolutionary change. For example, halving the play rate would double the duration of all stages of life history. For humans, this would mean an 18 month gestation time, puberty at ~28 years, adulthood at ~40, and a mean lifespan of ~170.

## 4 Further questions

The advances discussed here open up various other interesting new questions for future consideration. One relates to aging in organisms without DNA methylation. While vertebrate genomes are typically strongly methylated, those of invertebrates are either only very weakly methylated (de Mendoza et al., 2020), as in *D. melanogaster*, or not at all, as in the nematode *Caenorhabditis elegans*. Yet in *C. elegans*, as work from Anne Brunet’s lab has shown, alteration of determinants of epigenetic state can increase lifespan (Benayoun et al., 2015; Greer et al., 2011). These effects involve not DNA methylation but histone methylation (and acetylation).

This suggests that the function of methylation in mammalian epigenetic clocks may be the tip of a developmental-epigenetic iceberg; and that changes around those CpG sites are not binary but graded, involving changes in bivalent chromatin domains, with changes to histones as well as DNA. More broadly, the presence of DNA methylation-free developmental mechanisms of aging in *C. elegans* is in line with the view that the biological entity that determines mammalian aging rate is not the DNA methylation itself, but rather the developmental processes that it is a part of.

Another question relates to other kinds of aging. A form of aging where the role of developmental processes has long been accepted is that occurring in semelparous organisms that die rapidly as the result of extreme reproductive effort (reproductive death). These include Pacific salmon, lampreys and eels, and monocarpic plants, all of which exhibit DNA methylation. One possibility is that programmatic mechanisms present in iteroparous species become amplified during the evolution of semelparity, leading to rapid aging (Gems et al., 2021). It would be interesting to know whether epigenetic age is dramatically accelerated in semelparous organisms showing rapid reproductive death.

## 5 Conclusions

Recent years have witnessed exciting advances relating to methylation clocks and programmatic aging theory. We have highlighted here the convergence of these two paths of progress, and described several new ideas arising from this convergence. Understanding of the biological processes of aging that methylation clocks correspond to has lagged behind the recent rapid progress in clock characterization. Several features of clocks suggest that this hidden biology involves developmental processes. Recently developed programmatic theories offer a framework of ideas within which such developmental aging mechanisms can potentially be understood.

Such a framework includes explanations for how such clocks evolve, the programmatic mechanisms in which they act, and how those mechanism contribute to late-life disease. Central to this framework is the hypothesis that epigenetic and developmental changes occurring throughout life history are part and parcel of the same process.

We have also elaborated upon this explanatory framework, introducing new terminology (including the distinction between onto-developmental and maturo-developmental processes).

Hypotheses proposed include the regulation by polycomb proteins of a trade-off between developmental fidelity and plasticity, and the presence of a conserved developmental sequence as a major, previously unseen element in the aging process. We suggest that biological constraint imposed by both factors could be one determinant of the evolution of aging - of the length but not the play rate in the developmental sequence. These suggestions provide examples of the sort of thinking that is possible by combining ideas from evolutionary biology, biogerontology and developmental biology, in what we have described as a developmental gerontology or devo-gero approach. Such an approach can encompass not only the mechanisms of genetic determination of development and aging, but also determination by environmental factors affecting development in utero, such as maternal nutritional status, which can also influence health in later life (Fernandez-Twinn et al., 2019). Such effects may reflect either programmatic responses or disruption of normal biological processes; similarly, programmatic changes and disruptions to normal function contribute to late-life disease etiology (Gems, 2024). Notably, environmental factors likely to cause disruption (e.g. tobacco smoking, excessive alcohol consumption) can also increase estimated epigenetic age (Horvath and Raj, 2018). This could either reflect the fact that epigenetic clocks reflect general health status as well as adult developmental changes, or possibly an effect of disruption on such developmental changes during adulthood.
